# Supplementary material for: Polymer Pen Lithography-Fabricated DNA Arrays for Highly Sensitive and Selective Detection of Unamplified Ganoderma Boninense DNA
Source: Polymers (Basel). 2019 Mar 25;11(3):561. doi: 10.3390/polym11030561 (PMC6474127; doi:10.3390/polym11030561)
Supplement: Supplementary file 1 [file polymers-11-00561-s001.pdf]

Supplementary Materials

Scanning probe lithography based highly sensitive and selective detection of unamplified genomic DNA of *Ganoderma boninense*

Ekta Rani [1,2] Siti Akhtar Mohshim [3,4] Muhammad Zamharir Ahmad [4] Royston Goodacre [1,5] Shahrul Ainliah Alang Ahmad [3,6,*] and Lu Shin Wong [1,*]

[1] Manchester Institute of Biotechnology and School of Chemistry, University of Manchester, 131 Princess Street, Manchester M1 7DN, United Kingdom; ades.ekta@gmail.com (E.R.); roy.goodacre@liverpool.ac.uk (R.G.)
[2] Present address: Department of Physical Sciences, Central University of Punjab, Mansa Rd, 151001 Bathinda, Punjab, India
[3] Department of Chemistry, Universiti Putra Malaysia, 43400 Serdang, Selangor, Malaysia; ctakhtar@gmail.com
[4] Biotechnology and Nanotechnology Research Centre, Malaysian Agricultural Research and Development Institute, 43400 Serdang, Selangor, Malaysia; zamharir@mardi.gov.my
[5] Present address: Department of Biochemistry, Institute of Integrative Biology, University of Liverpool, Crown Street, Liverpool L69 7ZB, United Kingdom
[6] Institute of Advanced Technology, Universiti Putra Malaysia, 43400 UPM Serdang, Selangor, Malaysia
* Correspondence: l.s.wong@manchester.ac.uk (L.S.W.); ainliah@upm.edu.my (S.A.A.A.); Tel: +44-161306 8939 (L.S.W.); +60-389466805 (S.A.A.A.)

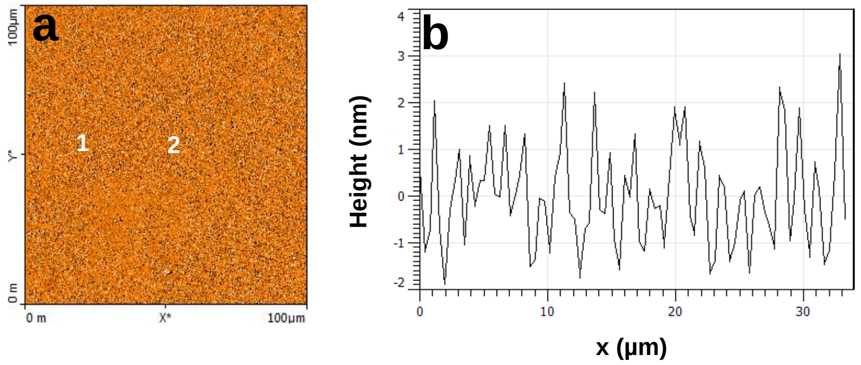

Supplementary Figure 1. (a) Representative AFM topography image of a substrate patterned with reporter DNA dissolved in DMF/aqueous solution (ink **1**), (b) representative corresponding cross-section profile along the line between points 1 and 2 marked in (a).

Number of copies of genomic DNA in the sample

The estimated number of copies of the target gene were calculated in the following manner. Given:

Avogadro's number, $N_A = 6.022 \times 10^{23}$ mol^{-1}

Average molecular weight of 1 bp (as sodium salt) = 650 Da

Number of base pairs in 1 copy of *G. boninense* genome[1], $BP_{genome} = 63.03 \times 10^6$ bp

Mass of genomic DNA in sample, m_{sample} = 400 ng = 4.0×10^{-7} g

Therefore:

Molecular weight of 1 copy of genome = $BP_{genome} \times 650$ = **4.10×10^{10} Da**

Number of moles of genome in sample = m_{sample} / (4.10×10^{10} Da) = **9.76×10^{-18} mol**

Number of copies of genome in sample = 2.44×10^{-19} mol $\times N_A$ = **5.88×10^6 copies**

And thus:

Number of copies of genome in 160 ng of genomic DNA sample = **2.35×10^6 copies**

Number of copies of genome in 30 ng of genomic DNA sample = **4.41×10^5 copies**

References

1. Mercière, M.; Laybats, A.; Carasco-Lacombe, C.; Tan, J. S.; Klopp, C.; Durand-Gasselin, T.; Syed Alwee, S. S. R.; Camus-Kulandaivelu, L.; Breton, F. Identification and development of new polymorphic microsatellite markers using genome assembly for Ganoderma boninense, causal agent of oil palm basal stem rot disease. *Mycol. Prog.* 2015, 14, 103.
